# Antidepressant Use in New-Onset Depression After Total Joint Arthroplasty Is Not Associated With Reduced Arthroplasty-Related Complications

**DOI:** 10.7759/cureus.81563

**Published:** 2025-04-01

**Authors:** Suin Jeong, Ji Won Lee, Elias K Shaya, Henry R Boucher

**Affiliations:** 1 Orthopaedics, MedStar Georgetown University Hospital, Washington, USA; 2 Orthopaedics, MedStar Union Memorial Hospital, Baltimore, USA; 3 Psychiatry, MedStar Good Samaritan Hospital, Baltimore, USA

**Keywords:** antidepressants, complications, new-onset depression, total hip arthroplasty, total knee arthroplasty

## Abstract

Background and objective

Some patients without pre-existing depression develop new-onset depression (NOD) following total joint arthroplasty (TJA), potentially impacting recovery and quality of life. New-onset depression has been associated with increased TJA complications (i.e., periprosthetic fracture, prosthetic joint infection, and revision), but the role of antidepressants in this population remains unexplored. This paper assessed the prevalence of antidepressant use in TJA patients with NOD and its association with postoperative complications.

Methods

We conducted a retrospective cohort study using a national database (2010-2022). Primary TJA patients aged ≥18 years with osteoarthritis and NOD within six months were included, while those with preoperative depression, antidepressant use, or < one-year follow-up were excluded. Study groups comprised those on antidepressants; controls consisted of those not on antidepressants. Logistic regressions adjusting for age, sex, and comorbidities assessed the odds of one-year postoperative complications.

Results

Among TJA patients with NOD, 25.3% (n=1,735) of total hip arthroplasty (THA) and 27.6% (n=4,365) of total knee arthroplasty (TKA) patients used antidepressants. Total hip arthroplasty patients on antidepressants had 1.70 times higher odds of periprosthetic fracture (95% CI: 1.22, 2.36) but showed no significant differences in prosthetic joint infection or revision. No significant differences were found in the TKA group.

Conclusions

Antidepressant use in NOD was not associated with reduced arthroplasty-related complications; however, treating depressive symptoms may still aid recovery. Further research, incorporating patient-level data on depression severity, therapy, and social support, antidepressant subclassification, and medication dosing and duration, is needed to identify which patients benefit from antidepressants and optimize postoperative mental health management.

## Introduction

Total joint arthroplasty (TJA) is an increasingly common procedure in the U.S. population, primarily indicated for patients with end-stage osteoarthritis, rheumatoid arthritis, post-traumatic arthritis, avascular necrosis, and other degenerative joint diseases that significantly impair function and quality of life. The number of primary total hip arthroplasty (THA) procedures is expected to grow to 635,000, and total knee arthroplasty (TKA) is projected to reach 1.26 million procedures by 2030 [1]. With the increasing prevalence of TJA, there is growing recognition of its impact on patients’ emotional homeostasis, particularly postoperative new-onset depression (NOD) in patients without a prior history of depression. New-onset depression may develop during the postoperative period [2-4], a time of anxiety and stress for patients undergoing TJA, triggered by post-surgical pain, limited mobility, prolonged recovery, and the psychological impact of surgery and adjusting to a prosthetic joint. While there is limited data on its prevalence, one U.S.-based national database study of 73,013 TJA patients without a history of depression in the year prior to surgery found 3.6% of them experienced NOD within six months [3]. Detecting new depressive symptoms following TJA in individuals without pre-existing depression may be more challenging compared to those with a known history of depression. Individuals with pre-existing depression may be more attuned to their symptoms and changes in their emotional state, enabling them to seek timely treatment, whereas those without a history of depression may fail to recognize their conditions promptly, potentially delaying diagnosis and treatment.

Identifying and managing depression is important because it may impact adherence to treatment and rehabilitation during the immediate postoperative period [5,6], which is essential for and may contribute to long-term TJA outcomes. Prior studies have found that TJA patients with pre-existing depression have lower patient-reported outcome and functionality scores, incur higher healthcare costs due to increased utilization (i.e., higher complication, non-home discharge, and readmission rates), and experience greater dissatisfaction compared to those without depression [7-9]. Additionally, a study on NOD found that TJA patients with NOD may be at risk for postoperative complications such as periprosthetic fracture, prosthetic joint infection (PJI), and revision surgery [10]. These findings demonstrate the importance of promptly recognizing and addressing depressive symptoms in postsurgical patients to optimize recovery and outcomes.

Antidepressants, such as selective serotonin reuptake inhibitors (SSRIs) and selective serotonin-norepinephrine reuptake inhibitors (SNRIs), are highly effective and widely prescribed for the treatment of depression [11,12]. To our knowledge, no studies to date have explored antidepressant use for NOD in TJA patients. However, there are studies on patients using antidepressants for pre-existing depression and TJA outcomes, although this evidence is mixed. While some studies on patients with pre-existing depression found antidepressant use to be associated with a lower risk of TJA revision [13], reduced opioid use after TKA [14], and similar improvement in physical function compared to non-depressed patients [15], one study found no association between depression treatment and patient-reported outcomes [16]. We wanted to explore whether these findings on antidepressants for pre-existing depression extended to NOD.

To address the gap in the literature, we examined the prevalence of antidepressant use among individuals with NOD within six months after TJA using a national administrative claims database. Our main aim was to investigate the association between antidepressant use in TJA patients with NOD and the odds of one-year arthroscopy-related complications.

## Materials and methods

Study design and setting

We performed a retrospective cohort analysis of the PearlDiver Mariner Patient Claims Database (PearlDiver Technologies, Colorado Springs, CO, USA). The PearlDiver database is a national Health Insurance Portability and Accountability Act-compliant database with over 161 million patients from January 2010 to April 2022 in its M161Ortho dataset. The data are de-identified, ensuring that patient-specific information is inaccessible. Only unique patient identifier codes are used to link data over time for longitudinal research, maintaining the confidentiality and security of all data in compliance with privacy regulations. All data processing was conducted within the PearlDiver database. All-payer claims (i.e., cash, commercial, government, Medicaid, and Medicare) in the database are adjudicated and audited by third parties. Using this database, we identified TJA patients who developed NOD within six months after surgery, excluding those with a pre-existing depression diagnosis or prior antidepressant use. We extracted data on antidepressant prescriptions, arthroplasty-related complications, and other relevant patient characteristics. Our analysis compared the rates of arthroplasty-related complications between patients who developed NOD and were newly prescribed antidepressants post surgery and those who developed NOD but were not started on antidepressants, adjusting for potential confounders such as age, sex, and comorbidities.

Sample

**Figure 1:**
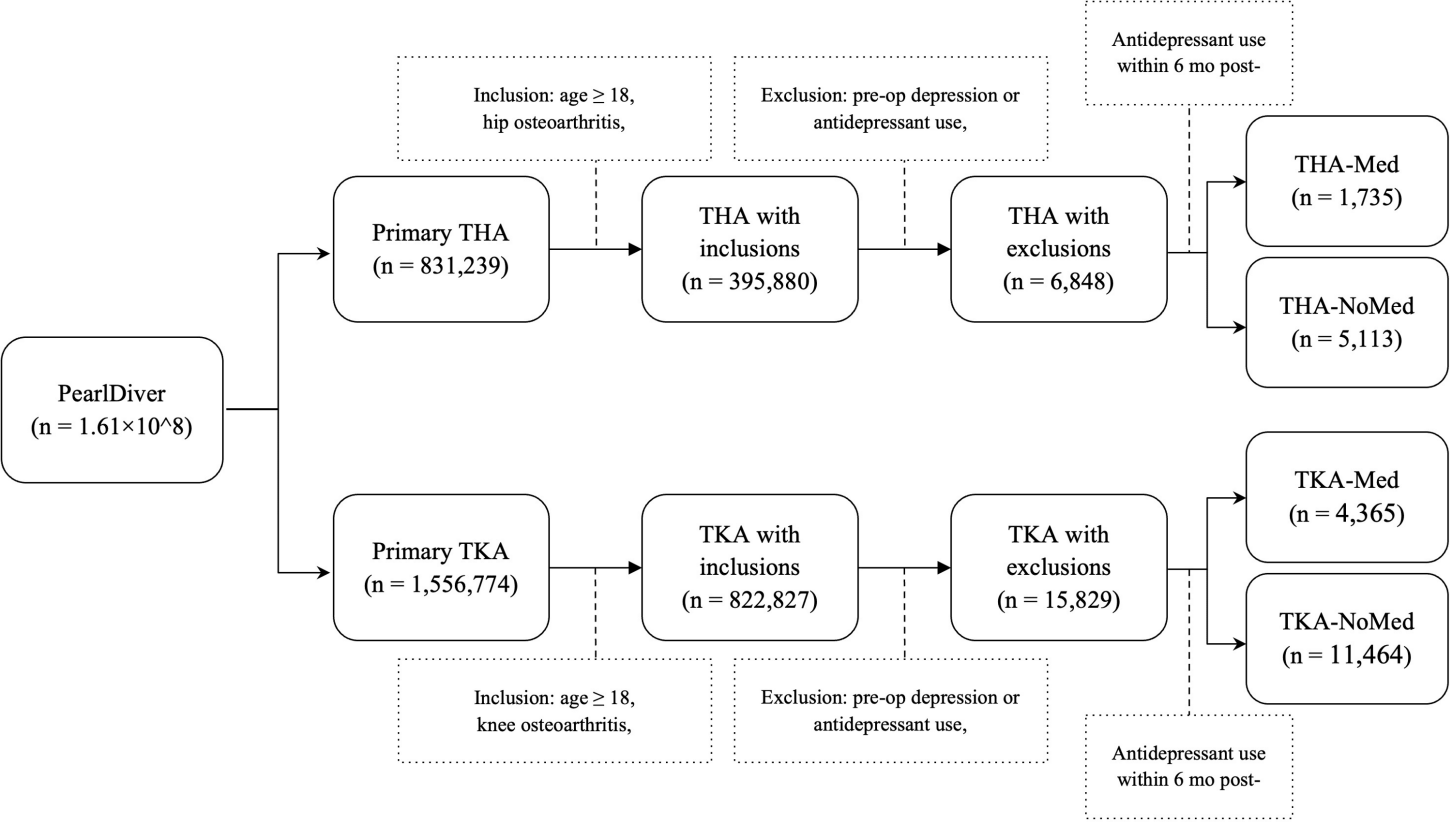
Flow diagram illustrating the inclusion and exclusion criteria used to define study participants Study groups included primary total hip arthroplasty (THA) and total knee arthroplasty (TKA) patients with new-onset depression who were started on antidepressants (THA-Med, TKA-Med). Control groups consisted of those who did not receive antidepressants (THA-NoMed, TKA-NoMed). pre-op: pre-operative; mo: months

Current Procedural Terminology (CPT), Uniform System of Classification (USC), and International Classification of Diseases-Ninth (ICD-9) [17] and Tenth revision (ICD-10) codes [18] were used to obtain our study and control groups (Appendix A). Patients were included if they underwent primary THA or TKA, had a history of hip or knee osteoarthritis, were a minimum of 18 years old, and developed NOD within six months of primary TJA. Those with a preoperative history of depression or antidepressant use and those who did not meet the minimum one-year study follow-up were excluded (Figure 1). Antidepressant use was identified using USC codes based on the PearlDiver database's categorization of the "antidepressant" class. This included SSRIs, SNRIs, tricyclic antidepressants, monoamine oxidase inhibitors, and atypical antidepressants such as bupropion and mirtazapine, as well as anxiolytics, mood stabilizers, and antipsychotic medications.

Of the total of 6,848 primary THA and 15,829 TKA patients with NOD, those started on antidepressants were identified as the study groups (THA, n=1,735; TKA, n=4,365). Patients with NOD not on antidepressants served as the controls (THA, n=5,113; TKA, n=11,464).

Ethical approval

This study was performed at MedStar Union Memorial Hospital in Baltimore, MD. This study was exempted from ethical approval by the institutional review board because the database was de-identified and there was no direct interaction with patients.

Outcome measures

The primary outcome was one-year postoperative surgical complications (i.e., periprosthetic fracture, PJI, and revision).

Data analyses

Demographic variables such as age, sex, and Charlson comorbidity index (CCI) were evaluated using descriptive statistics. Age and CCI were reported as median and interquartile ranges (IQR) since they displayed non-normality with the Shapiro-Wilk test. Sex was reported as counts and percentages. Wilcoxon and Chi-squared tests were used to compare continuous and categorical variables, respectively.

Logistic regression analyses were conducted on THA/TKA study and control group pairs, adjusting for age, sex, and CCI, which may be factors associated with new antidepressant use after surgery based on previous literature [19], to obtain the odds ratio (OR) with a 95% confidence interval (CI) for 1-year postoperative complications. Statistical significance was determined by a P-value <0.05. Analyses were conducted using the statistical software R (University of Auckland, Auckland, NZ) available within PearlDiver.

## Results

Baseline demographics

There were no statistically significant differences between the THA study and control groups in terms of age (66 versus 65 years, P=0.21), sex (61.2% versus 61.5% women, P=0.84), and CCI (one versus one, P=0.65) (Table 1). The TKA study group was older compared to the control group (67 versus 66 years, P<0.001); however, this difference was not clinically significant. The TKA group did not differ in sex (67.9% versus 68.9% women, P=0.26) and CCI (one versus one, P=0.12) (Table 1).

**Table 1:** Summary of patient demographics ^+^Wilcoxon test; *Chi-squared test Statistical significance was determined by a P-value <0.05. For THA patients with NOD, the test statistics values were as follows: age (W = 5849370), sex (Chi-squared 0.042), and CCI (W = 7939733). For TKA patients with NOD, the test statistics values were as follows: age (W = 26061511), sex (Chi-squared = 1.2901), and CCI (W = 41010365). CCI: Charlson Comorbidity Index; IQR: interquartile range; THA: total hip arthroplasty; TKA: total knee arthroplasty; NOD: new-onset depression

	Characteristics	Antidepressants	No antidepressants	P-value
THA patients with NOD	n	1,735	5,113	
	Age, median (IQR)	66 (57, 73)	65 (58, 72)	0.21^+^
	Women, n (%)	1,062 (61.2)	3,146 (61.5)	0.84*
	CCI, median (IQR)	1 (0, 2)	1 (0, 2)	0.65^+^
TKA patients with NOD	n	4,365	11,464	
	Age, median (IQR)	67 (59, 73)	66 (59, 72)	<0.001^+^
	Women, n (%)	2,966 (67.9)	7,899 (68.9)	0.26*
	CCI, median (IQR)	1 (0, 2)	1 (0, 2)	0.12^+^

Antidepressant use and one-year arthroplasty complications

Of THA patients with NOD, 25.3% (n=1,735) were started on antidepressant therapy. Of TKA patients with NOD, 27.6% (n=4,365) were started on antidepressant therapy.

The THA study group, compared to the control group, experienced 1.70 times higher odds of periprosthetic fracture (95% CI: 1.22, 2.36). However, there were no statistically significant differences in the odds of PJI (OR: 1.12, 95% CI: 0.85, 1.46) and revision surgery (OR: 1.24, 95% CI: 0.97, 1.57) (Table 2).

**Table 2:** One-year postoperative complications between total hip arthroplasty study and control groups Logistic regression analyses, adjusting for age, sex, and CCI, were conducted. Statistical significance was determined by a P-value <0.05. PJI: prosthetic joint infection; OR: odds ratio; CI: confidence interval; CCI: Charlson comorbidity index

Outcome	Antidepressants (n=1,735)	No antidepressants (n=5,113)	OR (95% CI)	P-value
Fracture, n (%)	57 (3.3)	100 (2.0)	1.70 (1.22, 2.36)	0.002
PJI, n (%)	78 (4.5)	206 (4.0)	1.12 (0.85, 1.46)	0.40
Revision, n (%)	101 (5.8)	243 (4.8)	1.24 (0.97, 1.57)	0.079

The TKA study group, compared to the control group, did not differ in the odds of periprosthetic fracture (OR: 1.04, 95% CI: 0.60, 1.74), PJI (OR: 0.95, 95% CI: 0.78, 1.14), and revision surgery (OR: 0.81, 95% CI: 0.63, 1.03) (Table 3).

**Table 3:** One-year postoperative complications between total knee arthroplasty study and control groups Logistic regression analyses, adjusting for age, sex, and CCI, were conducted. Statistical significance was determined by a P-value <0.05. PJI: prosthetic joint infection; OR: odds ratio; CI: confidence interval; CCI: Charlson comorbidity index

Outcome	Antidepressants (n=4,365)	No antidepressants (n=11,464)	OR (95% CI)	P value
Fracture, n (%)	19 (0.4)	48 (0.4)	1.04 (0.60, 1.74)	0.89
PJI, n (%)	148 (3.4)	410 (3.6)	0.95 (0.78, 1.14)	0.57
Revision, n (%)	90 (2.1)	290 (2.5)	0.81 (0.63, 1.03)	0.086

## Discussion

This study demonstrated that while 25.3% of THA and 27.6% of TKA patients with NOD were started on antidepressants, antidepressant use was not associated with differences in risk of arthroplasty-related complications. In fact, THA patients with NOD on antidepressants experienced higher odds of periprosthetic fracture compared to those not on antidepressants, but no difference was seen in the odds of PJI and revision. The TKA study and control groups did not differ in the odds of periprosthetic fracture, PJI, and revision.

To our knowledge, our study is the first to report the prevalence of antidepressant use among TJA patients with NOD and examine its association with arthroplasty-related complications. While NOD after TJA has been gaining attention recently [2-4], no prior studies have investigated whether management of this complication with antidepressants is associated with differences in arthroplasty-related complications. In TJA patients with NOD, our study suggests no overall difference in arthroplasty-related complications between antidepressant use and non-use, which contrasts with a prior study on pre-existing depression that reported a lower risk of TJA revision with antidepressant use [13]. However, unlike our study population, that newly developed depression post TJA, it is possible that patients with pre-existing depression had an established regimen with therapeutic effect prior to surgery, which may have positively affected postoperative complication rates.

Furthermore, there were higher odds of periprosthetic fracture among THA patients on antidepressants compared to those not on antidepressants in our study. Other risk factors for periprosthetic fracture, such as a history of osteoporosis prevalent among TJA patients [20] and the potential adverse effects of specific antidepressants [21-23], may have contributed to the development of postoperative complications, thereby confounding our results. For example, prior studies found SSRI use in the treatment of depression to be associated with higher risks of bone loss [21], falls [22], and fractures in the elderly, especially women [23], which may explain the higher odds of periprosthetic fracture in the THA study group compared to THA controls. Additionally, there is potential for bias, as patients on antidepressants may have had more severe depression, which was the reason they were started on antidepressants in the first place. Severe depression itself is a risk factor for poor health outcomes, including an increased likelihood of fractures, which could have further confounded our results. However, we also cannot rule out the possibility of reverse causation. Although we analyzed only complications that occurred after NOD, some patients may have already been experiencing symptoms such as persistent effusion and pain as early signs of postoperative complications before their formal diagnosis. These symptoms may have increased the likelihood of being prescribed antidepressants, leading to the observed positive association, rather than antidepressant use itself contributing to the development of complications.

Our findings have clinical and research implications. First, we report that over a quarter of TJA patients with NOD receive antidepressants. Our research highlights the importance of clearly demonstrating the indications and benefits of antidepressant use in this population, given that a significant portion of these patients are prescribed antidepressants. This will help guide the judicious and safe use of antidepressants, ensuring they are prescribed when most beneficial and appropriate for patients undergoing TJA. When strategizing pharmacological management, the benefits and risks of medications should be considered and weighed to achieve optimal outcomes, given that different antidepressants have different fall and fracture risk profiles [24]. Other management options, such as cognitive behavioral therapy [25], may be considered as an alternative or adjunct to medication management. Future studies may explore medications with both antidepressant and analgesic properties to see if they benefit those at risk for NOD. Studies have found that perioperative use of duloxetine, an SNRI, is associated with reduced pain in the immediate postoperative period and decreased opioid consumption, accelerating recovery and improving physical function [26]. A recent study also reported an association between perioperative esketamine administration and decreased postoperative depression scores and pain in elderly THA patients [27]. Further research on the benefits and risks of individual medications is crucial before they can be incorporated into practice for TJA patients with NOD, especially among older adults, and medication management should be tailored to individuals based on their demographics, comorbidities, and severity of depression.

Limitations

Limitations of this study include a lack of clinical data, such as depression severity, patient-reported TJA outcome measures, and patient satisfaction scores in the PearlDiver database, which limits a comprehensive evaluation of the association between antidepressant use and TJA outcomes. While all included patients developed depression within six months post surgery and continued to have a recorded diagnosis at one year postoperatively per ICD-9/10 codes [17, 18], database constraints prevented us from determining the exact duration of depressive episodes. We acknowledge this as a limitation and emphasize its importance for future studies. Some patients may also have had a remote, undiagnosed, or subclinical history of depression that was not captured by the database. Despite our efforts to exclude patients with a prior mental health history based on pre-existing depression diagnoses or prior antidepressant use, this limitation could have potentially confounded our results. Additionally, given that antidepressants generally require several weeks of treatment before a therapeutic benefit is realized [28], medication dosing/duration and adherence data may have influenced the degree of treatment effect, which our study did not include. We also did not examine specific types of antidepressants in our study. Future studies may include specific patient-level data on depression severity and duration, medication dosing/duration, and adherence, and subclassifications of antidepressants to enhance our understanding of antidepressants on long-term TJA outcomes. There are limited guidelines on when to start antidepressants for patients with NOD. Since we could not assess how long patients exhibited depressive symptoms or the severity of depression before being prescribed antidepressants, we are unable to determine which specific guidelines were followed, as this could have depended on patient and provider preferences, depression severity, and other factors. Nevertheless, we hope our findings provide valuable insights for future research and contribute to the development of guidelines for managing NOD in TJA patients.

Furthermore, while we attempted to adjust for relevant factors associated with antidepressant use and arthroplasty-related complications during our analyses, there may be unmeasured confounders, such as race, support systems, and the presence of psychotherapy, that we could not account for. Race/ethnicity, currently not available in the PearlDiver database, may be important to include in future studies because of the lower healthcare utilization rates for racial/ethnic minorities compared to White patients [29]. Studies have shown that race is associated with differences in antidepressant and psychotherapy utilization, with some groups experiencing lower treatment rates and longer delays in care compared to others [30]. Knowledge of the patient’s support system [31] and whether the patient received psychotherapy are also important confounders, as some patients may have undergone cognitive behavioral therapy [25] alone instead of medical management or received combination treatment. These factors could have differently influenced outcomes and further confounded our results. A recent systematic review examining the effect of depression interventions in TJA patients without a formal diagnosis of depression found that perioperative psychological optimization with psychotherapy and enhanced support are associated with improved depressive symptoms, pain, and function [32]. Therefore, race/ethnicity, as well as the presence of social support and therapy treatment, are important confounders that future studies can include. Despite these limitations, our study is the first to evaluate antidepressant use for NOD in TJA patients. While we did not find antidepressant use to be associated with differences in arthroplasty-related complications, antidepressants may still be beneficial for patients with severe and persistent depressive symptoms given their known efficacy in depression treatment [11,12].

Finally, as the study design does not involve direct interaction with patients, it may have been susceptible to bias, misinformation, or misinterpretation of information, which we attempted to minimize by carefully controlling for confounding variables and including/excluding patients based on the timing of when they developed depression, started antidepressants, and experienced complications.

## Conclusions

Our findings contribute to the growing body of research on the impact of NOD in TJA patients. Given the rising popularity of TJA, it is crucial to consider the impact of these procedures on patients’ emotional homeostasis. Monitoring and treating NOD may be essential in facilitating postoperative recovery. Despite the lack of established guidelines for managing depression in this context, our study found that antidepressants are frequently prescribed. Although we did not find antidepressant use among TJA patients with NOD to be associated with differences in arthroplasty-related complications, treating depressive symptoms may still offer benefits, especially for those with severe and persistent depression. Our findings have important implications for the development of future guidelines and clinical practice. By providing data on the prevalence of antidepressant use among TJA patients with NOD, our research lays the foundation for future studies focused on creating evidence-based recommendations for managing depression in this population. Future research is warranted to explore how to best manage NOD in TJA patients, whether with close surveillance or treatment (i.e., medications and/or therapy), and whether evidence could support the use of certain antidepressant medications over others based on their side effect profiles. This can lead to clearer guidelines on when to initiate antidepressant therapy, which specific medications to use, and how to monitor and adjust treatment to optimize both mental and physical recovery. As depression management remains a crucial component of comprehensive patient care, our study contributes to the evolving understanding of how antidepressants and other treatment modalities can be integrated into the care of TJA patients postoperatively, ultimately enhancing both their mental health and surgical outcomes.

## Appendices

Appendix A 

**Table 4:** CPT, ICD, and USC codes used CPT: Current Procedural Terminology; ICD-9/10: International Classification of Disease, Ninth/Tenth Revision; USC: Uniform System of Classification; THA: total hip arthroplasty; TKA: total knee arthroplasty

Description	Codes
Antidepressants	USC-64310, USC-64320, USC-64330, USC-64340, USC-64350, USC-64360, USC-64380, USC-64390
Depression	ICM-9: 296.20, 296.21, 296.22, 296.23, 296.24, 296.25, 296.26, 296.30, 296.31, 296.32, 296.33, 296.34, 296.35, 296.36, 296.82, 309.1, 311 ICM-10: F32.0, F32.1, F32.2, F32.3, F32.4, F32.5, F32.8, F32.89, F32.9, F33.0, F33.1, F33.2, F33.3, F33.4, F33.41, F33.42, F33.8, F33.9
Osteoarthritis of hip	ICD-9: 715.15, 715.25, 715.35, 715.95 ICD-10: M16.0, M16.10, M16.11, M16.12, M16.2, M16.30, M16.31, M16.32, M16.4, M16.50, M16.51, M16.52, M16.6, M16.7, M16.9
Osteoarthritis of knee	ICD-9: 715.16, 715.26, 715.36, 715.96 ICD-10: M17.0, M17.10, M17.11, M17.12, M17.2, M17.30, M17.31, M17.32, M17.4, M17.5, M17.9
THA	CPT: 27130
THA periprosthetic fracture	ICD-9: 996.44 ICD-10: M97.9XXA, T84.049A, M97.01XA, M97.02XA, T84.040A, T84.041A
THA prosthetic joint infection	ICD-9: 996.66 ICD-10: T84.50XA, T84.51XA, T84.52XA
THA revision	CPT: 27134, 27137, 27138
TKA	CPT: 27447
TKA periprosthetic fracture	ICD-9: 996.44 ICD-10: M97.9XXA, T84.049A, M97.11XA, M97.12XA, T84.042A, T84.043A
TKA prosthetic joint infection	ICD-9: 996.66 ICD-10: T84.50XA, T84.53XA, T84.54XA
TKA revision	CPT: 27486, 27487
